# Management of Crystallization Kinetics for Efficient and Stable Low‐Dimensional Ruddlesden–Popper (LDRP) Lead‐Free Perovskite Solar Cells

**DOI:** 10.1002/advs.201800793

**Published:** 2018-11-10

**Authors:** Jian Qiu, Yingdong Xia, Yonghua Chen, Wei Huang

**Affiliations:** ^1^ Key Laboratory of Flexible Electronics (KLOFE) & Institute of Advanced Materials (IAM) Jiangsu National Synergistic Innovation Center for Advanced Materials (SICAM) Nanjing Tech University (NanjingTech) 30 South Puzhu Road Nanjing 211816 P. R. China; ^2^ Shaanxi Institute of Flexible Electronics (SIFE) Northwestern Polytechnical University (NPU) 127 West Youyi Road Xi'an 710072 China; ^3^ Key Laboratory for Organic Electronics & Information Displays (KLOEID) and Institute of Advanced Materials (IAM) Nanjing University of Posts and Telecommunications 9 Wenyuan Road Nanjing 210023 China

**Keywords:** crystallization kinetics, lead‐free perovskites, Ruddlesden–Popper perovskites, stability

## Abstract

Low‐dimensional Ruddlesden–Popper (LDRP) lead‐free perovskite has great potential due to its improved stability and oriented crystal growth, which is mainly attributed to the effective control of crystallization kinetics. However, the crystallization kinetics of LDRP lead‐free perovskite films are highly limited by Lewis theory. Here, the management of the crystallization kinetics of LDRP tin (Sn) perovskite films jointly controlled by Lewis adducts and the ion exchange process using a mixture of polar aprotic solvent dimethyl sulfoxide (DMSO) and ion liquid solvent methylammonium acetate (MAAc) (the process named as “L‐I”) is demonstrated. Homogeneous nucleated LDRP Sn perovskite films with average grain size close to 9 µm are achieved. Both low electron and hole defect density with a magnitude of 10^16^, high carrier mobility, and excellent electrical performance are obtained. As a result, the LDRP Sn perovskite solar cell (PSC) with power conversion efficiency (PCE) of 4.03% is achieved using a simple one‐step method without antisolvents, which is one of the best LDRP Sn PSCs. Most importantly, the PSC exhibits excellent stability with no degradation in PCE after 94 d in a nitrogen atmosphere owing to the high‐quality film and the inhibition of the oxidation of Sn^2+^.

Organic–inorganic hybrid perovskite has aroused significant academic and industry attention because of its superior semiconducting properties, including high absorption coefficients, tunable bandgaps, high ambipolar charge mobility, low exciton binding energy, and long carries diffusion lengths.^1^, ^2^, ^3^, ^4^, ^5^, ^6^, ^7^, ^8^, ^9^, ^10^, ^11^, ^12^, ^13^, ^14^, ^15^, ^16^, ^17^ Much effort devoted in photovoltaic field, including material optimization, structural optimization, and interface engineering, has allowed the power conversion efficiency (PCE) of perovskite solar cells (PSCs) quick increase to 23.3%,^18^ which is comparable to state‐of‐the‐art commercial photovoltaic techniques, such as silicon and other thin‐film solar cells.

At present, in order to commercialize the perovskite solar cells, the research emphasis has shifted to develop the environment‐friendly, ultrastable, and large‐area PSCs. The toxic element lead (Pb) employed in traditional perovskite (e.g., CH_3_NH_3_PbI_3_) is an obstacle to the development of environment‐friendly perovskite photovoltaic devices. Many alternatives with lower toxicity have been proposed, involving Sn, Ge, Sb, Bi, and Cu etc.^19^ Among them, Sn‐based photovoltaic devices exhibit great potential because of its promising absorption caused by lower optical bandgap and room‐temperature stable crystal phase (α‐phase).^20^ Unfortunately, the low electrical resistivity on account of high p‐type doping caused by oxidation of Sn^2+^ to Sn^4+^ is a serious problem in Sn‐based perovskite, which results in the poor stability and low efficiency. Although great effort has been made in point view of materials with reducibility, such as SnF_2_,^21^ pyrazine,^22^ and triethylphosphine (TEP),^23^ and materials with hydrophobicity, such as phenylethylamine (PEA),^24^ to retard the oxidation of Sn^2+^ through Lewis acid‐base theory and vacuum evaporation technique to prevent the direct contact between Sn perovskite and ambient atmosphere,^25^, ^26^ problems remain.

To further improve the stability and efficiency of Sn‐based perovskite devices, the organic amine salts, such as butylamine (BA) and phenylethylamine (PEA), were introduced into the crystal lattice of Sn perovskite to form the low‐ dimensional Ruddlesden–Popper (LDRP) Sn perovskite, which have many advantages over 3D perovskite.^23^, ^27^ The introduction of different amount organic amine molecules will lead to the adjustment of the optical bandgap of perovskite for optimal energy level, which expand the application of lead‐free perovskite. Moreover, the crystallization process of perovskite films can be effectively regulated attributed to the introduction of organic amine molecules in LDRP lead‐free perovskite. Most importantly, the organic amine molecules can be self‐assembled with perovskite sheets through hydrogen bond so resulting in the quantum well structure with quantum confinement effect, which improve the thermodynamic stability and hydrophobic of lead‐free perovskite. The LDRP perovskites exhibited low self‐doping effect, suppressed ion migration, and excellent oriented growth over 3D perovskite due to this multiple‐quantum‐well structure,^27^, ^28^, ^29^, ^30^ which is benefit to inhibit the defect density in Sn perovskites. Kanatzidis and co‐workers^23^ for the first time demonstrated the LDRP Sn perovskite (BA)_2_(MA)*_n_*_−1_Sn*_n_*I_3_ *_n_*_+1_ devices with a best PCE of 2.5%. Furthermore, Ning et al.^27^ utilized the PEA to form the LDRP Sn perovskite with highly oriented crystal growth. The reported LDRP Sn perovskites have better stability than their 3D analogous. However, two‐step method with anti‐solvent was employed, which increased the complexity of the process.^24^, ^27^, ^31^ Furthermore, the investigation on controlling growth of high‐quality crystals with large domain by management of crystallization kinetics of Sn perovskite films has not been yet demonstrated.

Here, we for the first time reported the management of the crystallization kinetics of LDRP Sn perovskite jointly controlled by Lewis adducts and ion exchange process using mixture of polar aprotic solvent dimethyl sulfoxide (DMSO) and ion liquid solvent methyl‐ammonium acetate (MAAc) (name this process as “L‐I”). The homogeneous nucleated LDRP Sn perovskite film with ultralarge average grain size close to 9 µm was obtained owing to this dual regulation. The low charge carriers defect density with magnitude of 10^16^ and high carrier mobility were demonstrated. Based on the high quality films and low trap density, the LDRP Sn PSCs with PCE of 4.03% was obtained using simple one‐step method without antisolvents. Moreover, the PCE of devices with promising air stability and thermal stability has almost no decrease after 94 d in a nitrogen atmosphere. These results may provide in‐depth understanding of crystallization kinetics of LDRP Sn perovskite for promoting the development of high efficiency LDRP lead‐free PSCs with improved stability.

Briefly, we mixed the butylamine iodine (BAI), methyl ammonium chloride (MACl), SnI_2_, and SnF_2_ with proper stoichiometry in DMSO, MAAc, and their mixture (the volume ratio is 1:1), respectively, as shown in **Figure** **1**. The layer number of LDRP Sn perovskite was fixed at *n* = 4, that is, BA_2_MA_3_Sn_4_I_13_. The morphology of LDRP BA_2_MA_3_Sn_4_I_13_ perovskite films were first investigated by scanning electron microscopy (SEM) to understand the effect of different crystallization processes in film formation. As shown in Figure 1a, we clearly observed that the LDRP BA_2_MA_3_Sn_4_I_13_ perovskite films from DMSO (Lewis theory) have poor surface coverage, which is different from previous work demonstrating that the intermediate phase 3DMSO‐SnI_2_ formed by the Lewis base DMSO and SnI_2_ can retard the rapid reaction between SnI_2_ and MACl and achieve the high‐quality perovskite films.^3^ We proposed that this may be attributed to the introduction of BA cation which also has the effect of retarding the crystallization of perovskite,^28^, ^29^, ^32^ resulting in the excessive retardation of reaction between SnI_2_ and MACl and thus the poor surface coverage. When MAAc (ion exchange process) was used as precursor solvents, the intermediates BAMASn‐Ac was formed rapidly and then dense LDRP BA_2_MA_3_Sn_4_I_13_ perovskite films can be produced by ion exchange between I^−^ and Ac^−^ (Figure 1b).^13^ However, the films based on MAAc appeared the highest roughness of 31.906 nm (19.288 nm for DMSO based film) as a result of a large excess of MA^+^ (Figure S1, Supporting Information). Therefore, we combined the Lewis adduct and ion exchange process through the mixture of DMSO and MAAc (“L‐I”) and utilized this dual regulation to manage the crystallization kinetics of LDRP BA_2_MA_3_Sn_4_I_13_ perovskite (Figure 1c). As can be seen from Figure 1c, the film formed by means of “L‐I” process displayed not only high surface coverage, but also the lowest film roughness of 4.984 nm compared to Lewis theory process and ion exchange process (Figure S1, Supporting Information). Furthermore, ultralarge grain of LDRP BA_2_MA_3_Sn_4_I_13_ perovskite with average size of 9 µm was observed (Figure 1c and Figure S2 in Supporting Information), which is attributed to the homogeneous nucleation by “L‐I” process. We thus proposed that the larger perovskite crystals were achieved by effectively regulating the crystallization kinetics of LDRP Sn perovskite films through keeping balance between Lewis adducts and ion exchange process.

**Figure 1:**
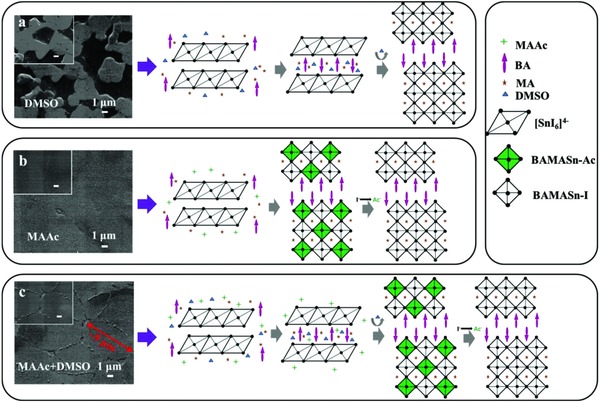
SEM images and schematic diagram of crystallization process of LDRP BA_2_MA_3_Sn_4_I_13_ perovskite films fabricated from different solvents: a) DMSO, b) MAAc, and c) DMSO+MAAc.

The LDRP BA_2_MA_3_Sn_4_I_13_ perovskite films prepared from different crystallization processes all showed diffraction peaks around 14.03° and 28.44° (**Figure** **2**a), corresponding to the (111) and (202) planes of perovskite, illustrating the unchanged perovskite lattice structure and the growth orientation perpendicular to the substrate by different crystallization processes.^23^, ^32^ Moreover, the narrowest FWHM (full width at half maximum, Figure S3 in Supporting Information) was obtained from mixed solvent system, demonstrating the best crystallinity in “L‐I” based LDRP BA_2_MA_3_Sn_4_I_13_ perovskite films, which could lead to the improved performance of the LDRP BA_2_MA_3_Sn_4_I_13_ perovskite devices (discussed below). In addition, the XRD pattern of LDRP perovskite films with *n* = 1 fabricating from “L‐I” process exhibited (0k0) planes corresponding to the previous report,^23^ which reasonably demonstrating the perovskite we prepared is LDRP phase perovskites (Figure S4, Supporting Information). To further investigate the effect of “L‐I” process on the LDRP BA_2_MA_3_Sn_4_I_13_ perovskite films, absorption and photoluminescence (PL) spectra were conducted (Figure 2b,c, and Figure S5, Supporting Information). The perovskite films based on DMSO and MAAc showed inconspicuous absorption band edges as a result of deep charge traps and high defect density (Figure 2b), which caused by poor film morphology leading to the oxidation of Sn^2+^.^31^ In contrast, the “L‐I” based films behaved the apparent absorption band edge at about 980 nm, indicating its higher quality film and better crystallinity, which is in agreement with the discussion above. Moreover, “L‐I” based films exhibit the higher PL intensity, which further demonstrated the low defect density. The red shift of PL occurred in MAAc and “L‐I” based film may be due to the excess MA^+^ (Figure 2c). The PL peaks of our LDRP Sn perovskite exhibited a red shift compared to PL peaks of single crystal BA_2_MA_3_Sn_4_I_13_,^23^ which was mainly due to the energy transfer from lower n phases to larger n phases in LDRP Sn perovskite with mixed phases fabricated by mixing raw materials with stoichiometric ratio (SnI_2_:MAI:BAI = 4:3:2).^33^, ^34^, ^35^, ^36^ Moreover, the PL spectra of LDRP Sn perovskite with different layers (n) and 3D Sn perovskite (Figure S5, Supporting Information) showed a blue shift of PL peaks from about 990 to 820 nm, corresponding to the phase from 3D to low dimension (LDRP, *n* = 2, 3, 4, etc.) and then to 2D (*n* = 1),^27^ which further demonstrates the existence of LDRP phase in our Sn perovskite fabricated from “L‐I” process.

**Figure 2:**
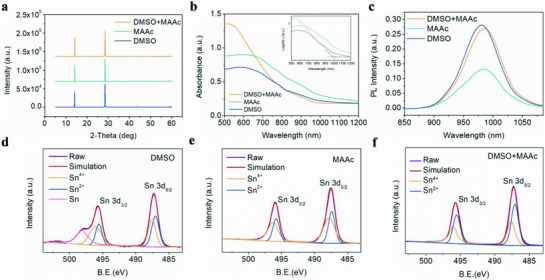
a) XRD pattern, b) Absorption spectra, and c) PL spectra of LDRP BA_2_MA_3_Sn_4_I_13_ perovskite films from different solvents. XPS spectrum of LDRP BA_2_MA_3_Sn_4_I_13_ perovskite films from different solvents: d) DMSO, e) MAAc, and f) DMSO+MAAc.

Generally, the defects or traps in Sn‐based perovskite are mainly attributed to the oxidation of Sn^2+^. In order to demonstrate this, X‐ray photoelectron spectroscopy (XPS) was used to compare the Sn^2+^ oxidation for the films from the DMSO, MAAc, and “L‐I” (Figure 2d–f). The fitting results illustrated that the perovskite films fabricated from “L‐I” process have the strongest Sn^2+^ peaks compared to Sn^4+^ peaks (Figure S6, Supporting Information), which indicated the high quality films formed by “L‐I” process can prevent the oxidation of Sn^2+^.

In order to further analyze the effect of “L‐I” process on the electron and hole trap density in LDRP BA_2_MA_3_Sn_4_I_13_ perovskite, the electron‐only device with structure of ITO/SnO_2_ (≈40 nm)/LDRP Sn perovskite (≈150 nm)/PCBM (≈80 nm)/LiF (1 nm)/Al (100 nm) and hole‐only device with structure of ITO/PEDOT:PSS (≈40 nm)/LDRP Sn perovskite (≈150 nm)/TFB (≈80 nm)/MoO_3_ (12 nm)/Al (100 nm) were fabricated. The electron trap‐state density and hole trap‐state density were calculated by dark current–voltage (*J*_D_ *–V*) curve of LDRP BA_2_MA_3_Sn_4_I_13_ perovskite prepared from DMSO, MAAc, and MAAc+DMSO using space‐charge‐limited‐current (SCLC) method. The trap‐state density was determined by the trap‐filled limit voltage using equation^37^

$$N_{t}=\frac{2\varepsilon_{0}\varepsilon_{r}V_{\mathrm{TFL}}}{qL^{2}}$$

where *L* is the thickness of LDRP BA_2_MA_3_Sn_4_I_13_ perovskite film, ε_r_ (= 25) is relative dielectric constant, ε_0_ is the vacuum permittivity, *q* is the elemental charge, and *V*_TFL_ is the onset voltage of the trap filled limit region (**Figure** **3**, TFL). All data are summarized in **Table** **1**. As shown in Figure 3 and Table 1, “L‐I” process demonstrated ten times order of magnitude lower than others (10^16^ vs 10^17^). Such a low trap state density is mainly attributed to improved quality LDRP BA_2_MA_3_Sn_4_I_13_ perovskite films with ultralarge grain size reducing the grain boundary and oxidation of Sn^2+^. Moreover, the intrinsic electron and hole mobilities were estimated via the same method using the Mott‐Gurney Law^37^

$$J_{D}=\frac{9\varepsilon \;\varepsilon_{0}\;\mu V_{b}{}^{2}}{8L^{3}}$$

**Figure 3:**
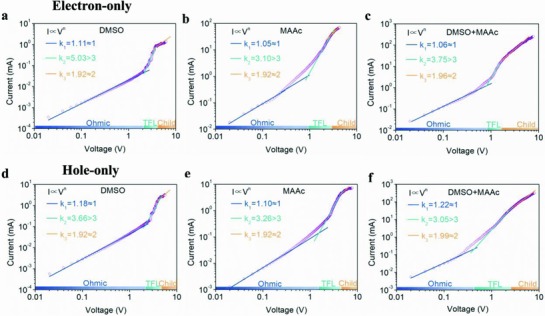
Dark current–voltage curves of electron‐only devices for LDRP BA_2_MA_3_Sn_4_I_13_ perovskite from different solvents, a) DMSO, b) MAAc, and c) DMSO+MAAc. Dark current–voltage curves of the hole‐only devices for LDRP BA_2_MA_3_Sn_4_I_13_ perovskite from different solvents, d) DMSO, e) MAAc, and f) DMSO+MAAc.

**Table 1:** Electron and hole trap density and charge carriers mobilities of LDRP BA_2_MA_3_Sn_4_I_13_ perovskite films from different processes

Different processes	*N*_t(e)_ [cm^−3^]	*N*_t(h)_ [cm^−3^]	*µ*_e_ [cm^2^ V^−1^ s^−1^]	*µ*_h_ [cm^2^ V^−1^ s^−1^]
DMSO	2.7 × 10^17^	2.6 × 10^17^	5.2 × 10^−5^	1.1 × 10^−4^
MAAc	1.1 × 10^17^	1.9 × 10^17^	4.4 × 10^−3^	2.2 × 10^−4^
DMSO+MAAc	9.8 × 10^16^	5.4 × 10^16^	7.2 × 10^−3^	6.8 × 10^−3^

The *V*_b_ and *J*_D_ in the equation are defined as applied voltage and applied current, respectively. The calculation results show that BA_2_MA_3_Sn_4_I_13_ perovskite devices fabricated from “L‐I” process possess not only higher electron mobility (7.2 × 10^−3^ cm^2^ V^−1^ s^−1^) but also higher hole mobility (6.8 × 10^−3^ cm^2^ V^−1^ s^−1^) compared to that of Lewis theory process (5.2 × 10^−5^ cm^2^ V^−1^ s^−1^ for electron and 1.1 × 10^−4^ cm^2^ V^−1^ s^−1^ for hole) and ion exchange process (4.4 × 10^−3^ cm^2^ V^−1^ s^−1^ for electron and 2.2 × 10^−4^ cm^2^ V^−1^ s^−1^ for hole) (Figure 3 and Table 1). The highest charge carrier mobility of “L‐I” based BA_2_MA_3_Sn_4_I_13_ perovskite due to the low trap density and high quality films are in good agreement with the pervious results.

Having shown the improved optoelectronic properties of LDRP BA_2_MA_3_Sn_4_I_13_ perovskite from “L‐I” process, we fabricated the LDRP BA_2_MA_3_Sn_4_I_13_ PSCs with the structure of ITO/PEDOT:PSS (≈40 nm)/perovskite (≈150 nm)/PCBM (≈80 nm)/LiF (1 nm)/Al (100 nm). The performance was evaluated by measuring their photocurrent density versus voltage (*J–V)* curves under the simulated air mass 1.5 global (AM1.5G) solar irradiation. As shown in **Figure** **4**a and **Table** **2**, the maximum PCE of 4.03% with open‐circuit voltage (*V*_oc_) of 0.38 V, short‐circuit current (*J*_sc_) of 21.87 mA cm^−2^, and fill factor (FF) of 48.30% was achieved from “L‐I” process, whereas DMSO and MAAc based devices gave PCEs of 0.44% and 2.08%, respectively. Furthermore, the PCE of our LDRP perovskites is much higher than that of 3D counterparts (Figure S7, Supporting Information). The high *V*_oc_ and *J*_sc_ observed in “L‐I” based device are mainly due to the high quality films and ultralarge grain size. The dense and smooth films with no pinhole are known to be able to decrease or mitigate the bulk defect, which reduced charge recombination, improved charge transport, and promoted charge extraction. As shown in Figure 4b, the device fabricated from “L‐I” process showed the lowest background carrier concentration, which, on one hand, indicated the lower p‐doping level and lower trap state density attributed to the inhibition of oxidation of Sn^2+^ compared to that of devices from DMSO and MAAc processes, corresponding to the results of XPS (Figure 2d–f) and SCLC (Figure 3); on the other hand, demonstrated the significantly reduced charge recombination. Moreover, as shown in Figure S8 (Supporting Information), the *V*_oc_ versus light intensity characterization also confirm that the reduced charge recombination^38^ and the lowered trap density of the “L‐I” device (*n* = 0.094) compared to those of DMSO (*n* = 0.153) and MAAc (*n* = 0.101) devices, resulting in the effective transfer and extraction of charge. In addition, the charge transport resistances of devices based on different processes were detected via electrical impedance spectrum (Figure 4c). The charge transport resistance of “L‐I” device (≈1236 Ω cm^2^), which is lower than those of DMSO (≈5879 Ω cm^2^) and MAAc (≈9300 Ω cm^2^) devices, conformed again the improved charge transport corresponding to the high carrier mobility (Figure 3). Most importantly, the devices prepared from “L‐I” process show almost no hysteresis effect with different scanning directions or voltage delay times (Figure 4d and Figure S9, Supporting Information) and behave stable current and power output (Figure 4e) whereas the hysteresis effect and unstable output are obvious in previous reports,^39^, ^40^ indicating that the efficiencies measured for the devices with stable *J–V* curves are reliable. In general, the hysteresis effect and unstable output mainly originated from the traps and defects, which can capture the carriers.^41^ Therefore, the ultralow hysteresis effect and stable output in our device further demonstrated the lower trap density in the high quality films fabricated from “L‐I” process. The other possible contribution of interface passivation of PCBM cannot be ruled out as previous reports.^42^, ^43^ The PCE statistics of total 45 devices (DMSO, MAAc, and “L‐I” based devices) are summarized in Figure 4f, illustrating that high‐performance PSCs can be repeatedly fabricated by “L‐I” process. Impressively, the PCE of LDRP BA_2_MA_3_Sn_4_I_13_ PSCs fabricated from “L‐I” process showed almost no decrease in N_2_ for 94 d (Figure 4g), which is one of the most stable devices as far as we know among the reported Sn‐based PSCs (Table S1, Supporting Information). The unprecedented stability can be attributed to the dense and smooth LDRP BA_2_MA_3_Sn_4_I_13_ perovskite films fabricated from “L‐I” process possessing the capability to prevent the oxidation of Sn^2+^. Moreover, we fabricated the LDRP BA_2_MA_3_Sn_4_I_13_ perovskite devices in air and achieved the PCE of 1.48% (Figure S10, Supporting Information), which has previously suggested to be difficult. Most importantly, the LDRP BA_2_MA_3_Sn_4_I_13_ perovskite films fabricated from “L‐I” process exhibited no SnI_2_ peaks, but only a slight decrease in crystallinity when stored in external environment for 285 min and heating environment (85 °C in N_2_) for 40 min (Figure S11, Supporting Information). These findings indicate that our LDRP BA_2_MA_3_Sn_4_I_13_ perovskite based on “L‐I” process has a great potential in the future application.

**Figure 4:**
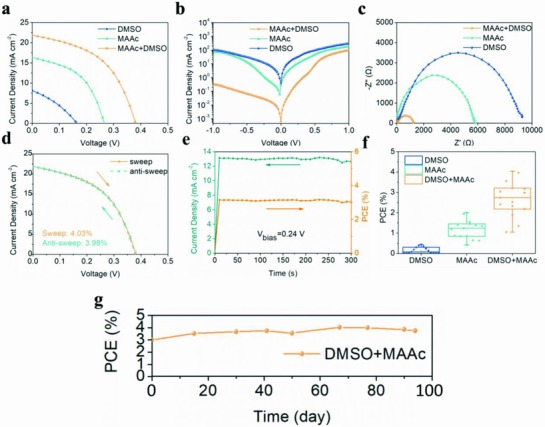
a) Photocurrent density versus voltage (*J–V)* curves, b) dark *J–V* curves, and c) electrical impedance spectrum of LDRP BA_2_MA_3_Sn_4_I_13_ perovskite films from different solvents. d) Hysteresis effect test of LDRP BA_2_MA_3_Sn_4_I_13_ perovskite devices based on “L‐I” process. e) Stable output of LDRP BA_2_MA_3_Sn_4_I_13_ perovskite device at *V*_bis_ = 0.24 V for 300 s. f) The PCE distribution statistics of the studied devices. g) The stability of LDRP BA_2_MA_3_Sn_4_I_13_ perovskite device stored in N_2_ atmosphere.

**Table 2:** Summary of device parameters from different processes

Different process	*V*_oc_ [V]	*J*_sc_ [mA cm^−2^]	FF [%]	PCE [%]
DMSO	0.16	8.14	32.72	0.44
MAAc	0.26	16.37	47.86	2.08
DMSO+MAAc	0.38	21.87	48.30	4.03

In conclusion, the coregulation of Lewis adduct and ion exchange process was introduced to control the crystallization kinetics of LDRP BA_2_MA_3_Sn_4_I_13_ perovskite films using mixture of DMSO and MAAc. Owing to the effect of this management, the dense and smooth LDRP BA_2_MA_3_Sn_4_I_13_ perovskite films with ultralarge grain size close to 9 µm, high carrier mobility, and low defect density with magnitude of 10^16^ were obtained. Finally, we achieved PSCs with PCE of 4.03% in N_2_ atmosphere and 1.48% in air utilizing the simple one‐step method without antisolvents. Most impressively, the PCE appeared almost no decrease in N_2_ for 94 d, indicating the excellent stability. These results will offer in‐depth understanding of crystallization kinetics of LDRP Sn based perovskite to promote the development of high efficiency lead‐free PSCs with improved stability.

## Experimental Section

*Materials and Preparation of BAI and MAAc*: PEDPT:PSS (Clevious PVP. AL 4083) was purchased from Heraeus‐(Deutschland GmBH & Co. KG) Inc. PCBM was purchased from Xi'an Polymer Light Technology Corp. Tin iodide (SnI_2_, purity ≥99.999%) was purchased from Alfa Aesar and tin fluoride (SnF_2_, purity >95%) was purchased from Sigam Aldrich. Methylammonium chloride (CH_3_NH_3_Cl, purity>99%) was purchased from Shanghai MaterWin New Materials Co. Ltd. DMSO, *n*‐butylamine, hydroiodic acid (HI) (57 wt% in water), and acetic acid (HAc) (99%) were got from Sigma‐Aldrich. Methylamine (MA, 33% in water) was purchased from Energy Chemical.

*BAI*: BA and HI were mixed in anhydrous ethanol at a molar ratio of 1:1.1 and then were stirred under ice bath for 2 h. The solution was then evaporated to crystallize and the resulting crystals were washed using ether. After recrystallization, the BAI was obtained by drying.

*MAAc*: MA and HAc were mixed at a molar ration of 1:1 and then stirred under ice bath for 2 h. The obtained solution was then evaporated until no anhydrous ethanol and washed using ether.

*Precursor Solution*: BAI, MACl, SnI_2_, and SnF_2_ were mixed in DMSO, MAAc and mixture of them (v:v = 1:1) at a molar ratio of 2:3:4:0.2 and then stirred for 2 h.

*Perovskite Film Deposition*: The precursor was preheated at 60 °C and then spin coated on PEDOT:PSS at 4000 r.p.m. for 20 s with 70 °C in N_2_ or air. Then the films were annealed at 60 °C for 1 min and 100 °C for 4 min (N_2_).

*Fabrication*: ITO glass was cleaned and then treated by UV ozone for 15 min before use. PEDOT:PSS was spin coated at 5000 r.p.m. for 50 s and then annealed at 120 °C for 30 min. Then perovskite was spin coated on them. After that, PCBM (18 mg in 1 mL chlorobenzene) was spin coated at 1000 r.p.m. for 60 s and 2000 r.p.m. for 2 s. Finally, LiF (1 nm) and Al (100 nm) were evaporated under high vacuum. For device test, the device area is defined as 0.05 cm^2^.

*Characterization*: SEM images were obtained from Hitachi S‐4300 and the instrument used an electron beam accelerated at 10–30 kV, enabling operation at a variety of currents. AFM images were obtained from ThermoMicroscope M5 in non‐contact mode. The XRD pattern was obtained using an X‐ray diffractometer, Bruker AXS D8, with Cu Kα radiation. The XPS spectrum was measured by a Kratos Axis Ultra DLD photoelectron spectrometer. The current–voltage (*J–V*) curves of the devices were measured in glovebox using a Keithley 2400 source meter and the solar cells with effective area was 0.05 cm^2^ were illuminated under 100 mW cm^−2^ Air Mass 1.5 global (AM 1.5G). The PL spectrum was obtained from fluorescent spectrometer (HORIBA FluoroLog‐3) and the absorption spectrum was obtained from Cary 5000 UV–vis–NIR.

## Conflict of Interest

The authors declare no conflict of interest.

## Supporting information

Supplementary
