# Supplementary material for: Management of Crystallization Kinetics for Efficient and Stable Low‐Dimensional Ruddlesden–Popper (LDRP) Lead‐Free Perovskite Solar Cells
Source: Adv Sci (Weinh). 2018 Nov 10;6(1):1800793. doi: 10.1002/advs.201800793 (PMC6325589; doi:10.1002/advs.201800793)
Supplement: Supplementary file 1 — Supplementary [file ADVS-6-1800793-s001.pdf]

Copyright WILEY-VCH Verlag GmbH & Co. KGaA, 69469 Weinheim, Germany, 2018.

ADVANCED SCIENCE
Open Access

Supporting Information

for *Adv. Sci.,* DOI: 10.1002/advs.201800793

Management of Crystallization Kinetics for Efficient and Stable Low-Dimensional Ruddlesden–Popper (LDRP) Lead-Free Perovskite Solar Cells

Jian Qiu, Yingdong Xia, Yonghua Chen, and Wei Huang**

Supporting Information

Management of crystallization kinetics for efficient and stable low dimensional Ruddlesden-Popper (LDRP) lead-free perovskite solar cells

Jian Qiu,[1] Yingdong Xia,[1] Yonghua Chen,[1]* Wei Huang[1,2,3]*

[1]Key Laboratory of Flexible Electronics (KLOFE) & Institute of Advanced Materials (IAM), Jiangsu National Synergistic Innovation Center for Advanced Materials (SICAM), Nanjing Tech University (NanjingTech), 30 South Puzhu Road, Nanjing 211816, P.R. China. [3]Shaanxi Institute of Flexible Electronics (SIFE), Northwestern Polytechnical University (NPU), 127 West Youyi Road, Xi'an 710072, China. [4]Key Laboratory for Organic Electronics & Information Displays (KLOEID), and Institute of Advanced Materials (IAM), Nanjing University of Posts and Telecommunications, 9 Wenyuan Road, Nanjing 210023, China.

*Corresponding author. E-mail: iamyhchen@njtech.edu.cn; iamwhuang@njtech.edu.cn

J.Q. and Y.X. contributed equally to this work.

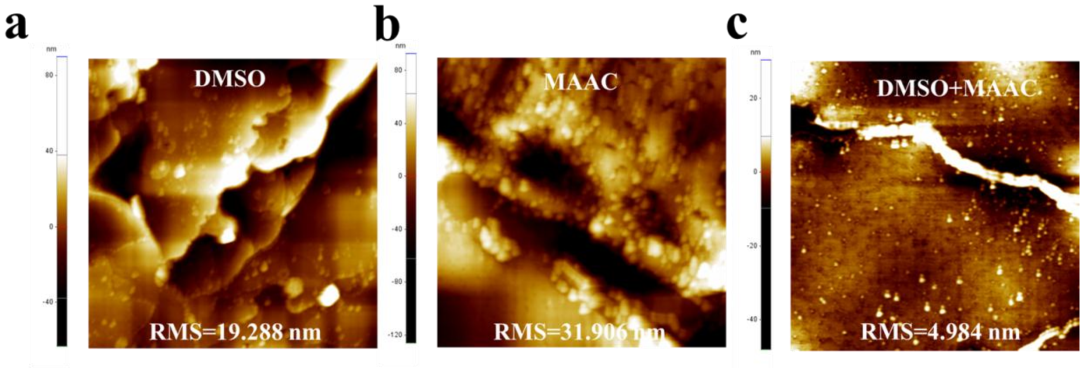

Figure S1. AFM images of LDRP $BA_2MA_3Sn_4I_{13}$ perovskite films from different crystallization processes: (a) DMSO, (b) MAAc, and (c) DMSO+MAAc.

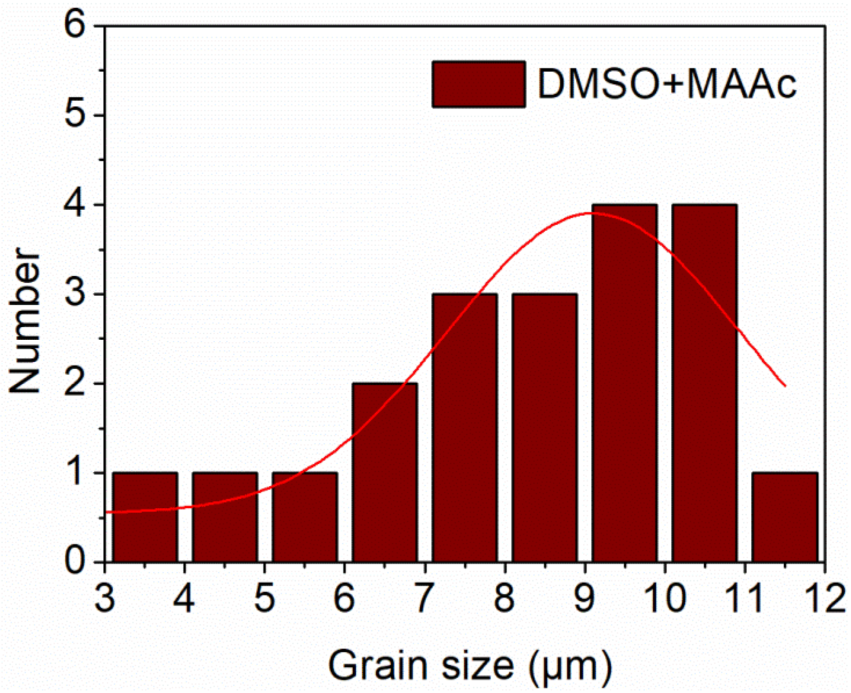

Figure S2. Grain size distributions of LDRP $BA_2MA_3Sn_4I_{13}$ perovskite films from DMSO+MAAc solvent.

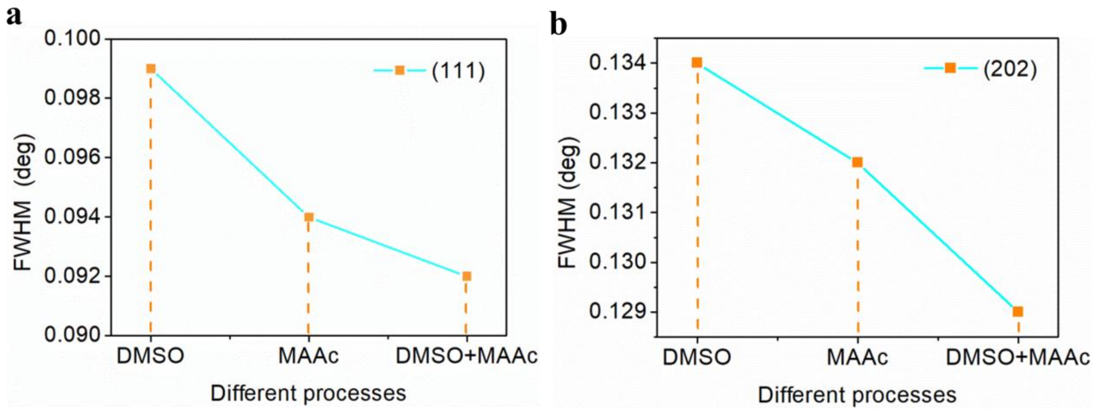

Figure S3. Full width at half maximum (FWHM) of XRD pattern from different processes: (a) peak (111), and (b) peak (202).

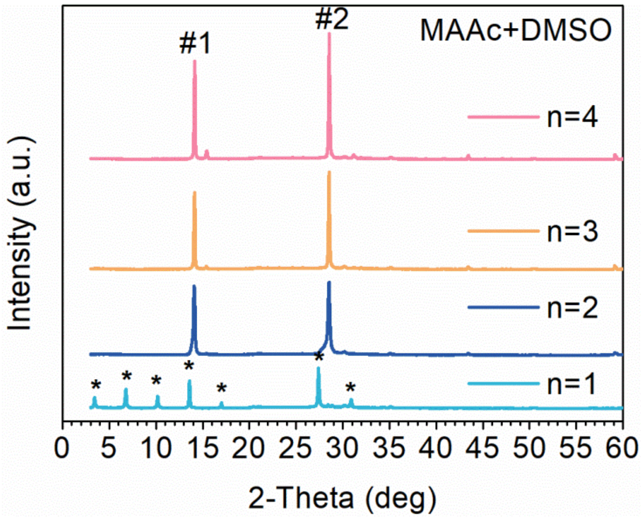

Figure S4. The XRD pattern of LDRP Sn perovskite films with different layers (n) from 'L-I' process.

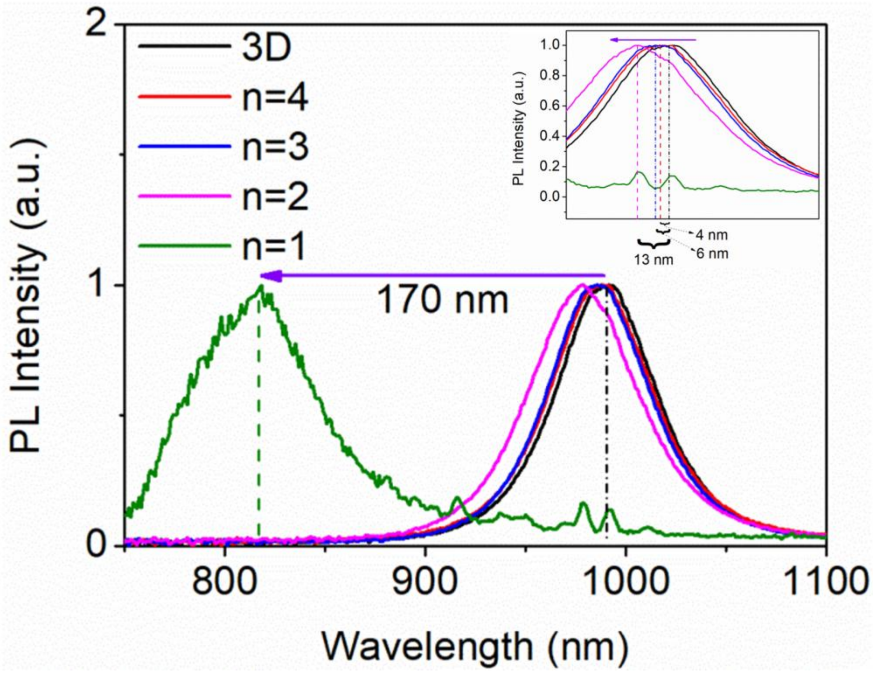

Figure S5. The PL spectra of LDRP Sn perovskite films with different layers (n) and 3D Sn perovskite films.

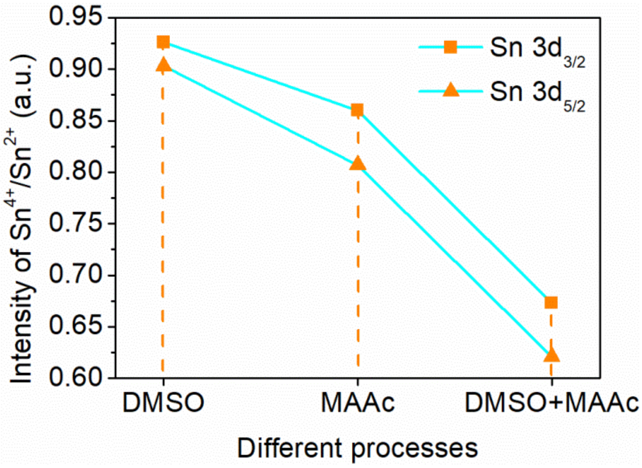

Figure S6. Comparison of intensity of Sn^{4+}/Sn^{2+} in LDRP $BA_2MA_3Sn_4I_{13}$ perovskite films from different crystallization processes.

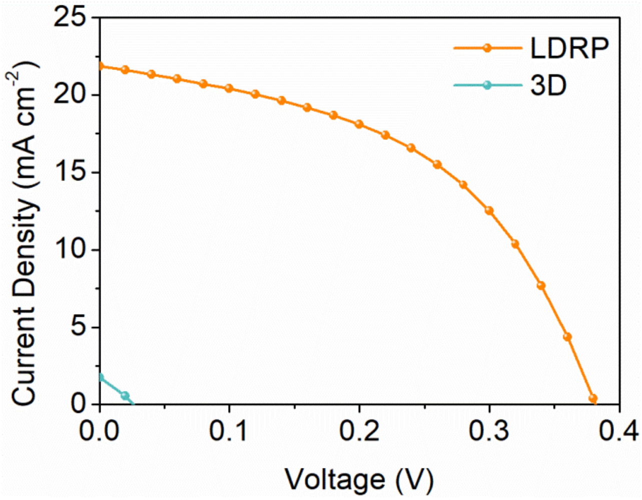

Figure S7. Photocurrent density versus voltage (*J-V)* curves of LDRP and 3D perovskite devices fabricated from 'L-I' process.

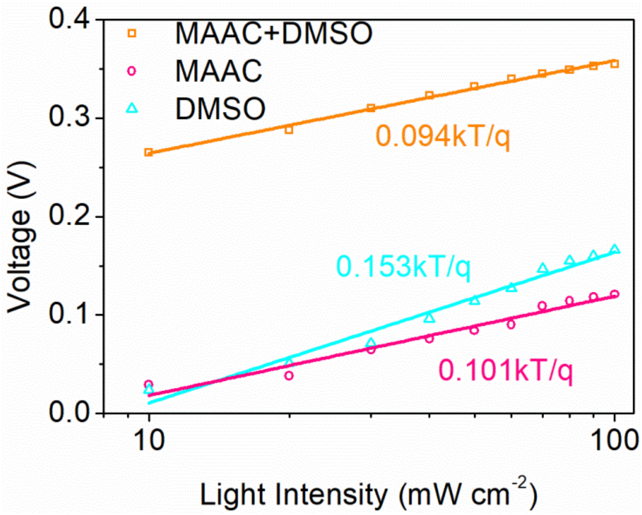

Figure S8. V_{oc} *VS* light intensity characterization of LDRP $BA_2MA_3Sn_4I_{13}$ perovskite devices based on different processes.

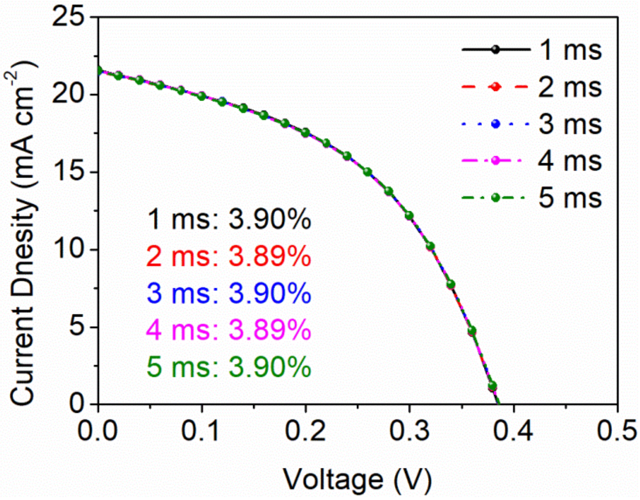

Figure S9. Hysteresis characteristics of LDRP $BA_2MA_3Sn_4I_{13}$ PSCs fabricated from 'L-I' process.

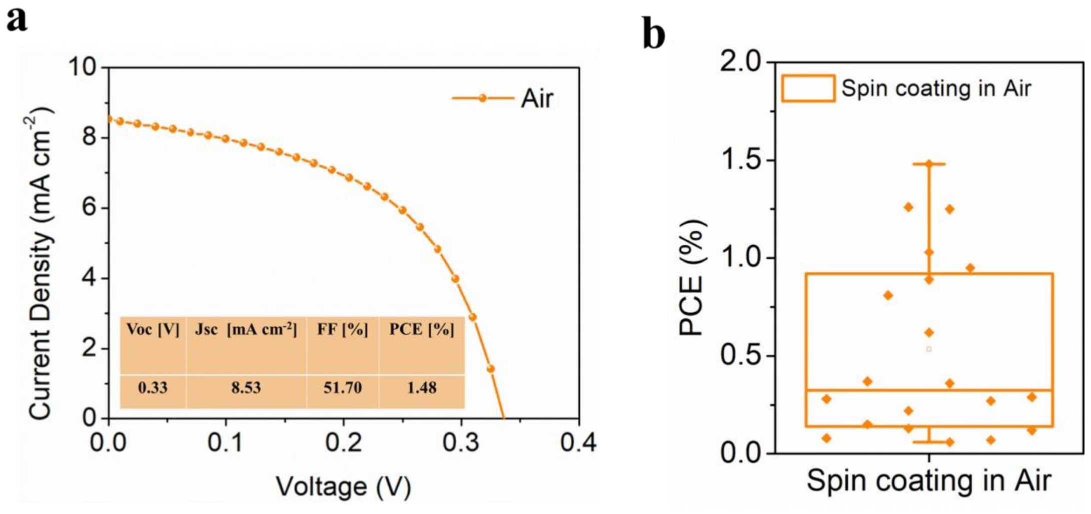

Figure S10. (a) The PCE of LDRP $BA_2MA_3Sn_4I_{13}$ PSCs fabricated in air and (b) the statistics of PCEs of the studied devices.

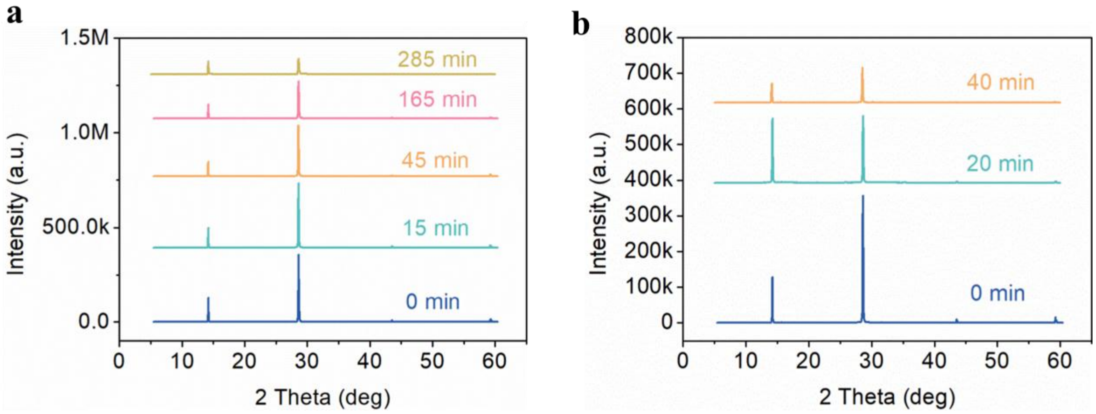

Figure S11. Stability of LDRP $BA_2MA_3Sn_4I_{13}$ perovskite films fabricated from 'L-I' process: (a) in air, and (b) at 85 ℃ in N_2 atmosphere.

Table S1. The stability of the state-of-art Sn-based PSCs.

Perovskites	Time	Maintain of initial PCE	Atmosphere	References
$MASnI_3$	1 day	36%	N_2	[1]
$BA_2MA_3Sn_4I_{13}$	30 day	93%	N_2	[2]
$MASnIBr_{1.8}Cl_{0.2}$	83 day	90%	N_2	[3]
$FA_{0.75}MA_{0.25}SnI_3$	16 day	80%	N_2	[4]
$CsSnI_3$	11 day	＞95%	N_2	[5]
$CsSnI_3$	10 day	＞85%	N_2	[6]
$CsSnI_3$	0.6 day	70%	Air	[7]
$CsSnI_3$ (QR)	16 day	54%	N_2	[8]
$CsSnBr_3$	0.03 day	＞95%	Air	[9]
$FASnI_3$	25 day	＞95%	N_2	[10]
$FASnI_3$	20 day	＞80%	N_2	[11]
$FASnI_3$	6 day	31%	N_2	[12]
$FASnI_3$	30 day	85%	N_2	[13]
$FASnI_3$	100 day	98%	N_2	[14]
$PEA_2FA_8Sn_9I_{28}$	4 day	＞95%	N_2	[15]
0.08PEA-0.92FA SnI_3	3 day	59%	Air	[16]
2D-3D bulk heterojunction PEA-$FASnI_3$	5 day	90%	N_2	[17]
$BA_2MA_3Sn_4I_{13}$	94 day	＞99%	N_2	This work

References

[1] F. Hao, C. C. Stoumpos, D. H. Cao, R. P. H. Chang, M. G. Kanatzidis, *Nat. Photonics* **2014**, *8*, 489.

[2] D. H. Cao, C. C. Stoumpos, T. Yokoyama, J. L. Logsdon, T. Song, O. K. Farha, M. R. Wasielewski, J. T. Hupp, M. G. Kanatzidis, *ACS Energy Lett.* **2017**, *2*, 982.

[3] E. W. Diau, C. Tsai, N. Mohanta, C. Wang, Y. Lin, Y. Yang, C. Wang, C. Hung, *Angew. Chem. Int. Ed.* **2017**, *129*, 14007.

[4] Z. Zhao, F. Gu, Y. Li, W. Sun, S. Ye, H. Rao, Z. Liu, Z. Bian, C. Huang, *Adv. Sci.* **2017**, *4*, 170020.

[5] M. Kumar, S. Dharani, W. Leong, P. P. Boix, R. R. Prabhakar, T. Baikie, C. Shi, H. Ding, R. Ramesh, M. Asta, M. Graetzel, S. G. Mhaisalkar, N. Mathews, *Adv. Mater.* **2014**, *26*, 7122.

[6] K. P. Marshall, R. I. Walton, R. A. Hatton, *J. Mater. Chem. A* **2015**, *3*, 11631.

[7] K. P. Marshall, M. Walker, R. I. Walton, R. A. Hatton, *Nat. Energy* **2016**, *1*, 16178.

[8] L. Chen, C. Lee, Y. Chuang, Z. Wu, C. Chen, *J. Phys. Chem. Lett.* **2016**, *7*, 5028.

[9] D. Moghe, L. Wang, C. J. Traverse, A. Redoute, M. Sponseller, P. R. Brown, V. Bulović, R. R. Lunt, *Nano Energy* **2016**, *28*, 469.

[10] S. J. Lee, S. S. Shin, J. Im, T. K. Ahn, J. H. Noh, N. J. Jeon, S. I. Seok, J. Seo, *ACS Energy Lett.* **2018**, *3*, 46.

[11] Z. Zhu, C.Chueh, N. Li, C. Mao A. K.-Y. Jen, *Adv. Mater.* **2017**, *30*, 1703800.

[12] J. Xi, Z. Wu, B. Jiao, H. Dong, C. Ran, C. Piao, T. Lei, T. Song, W. Ke, T. Yokoyama, X. Hou, M. G. Kanatzidis, *Adv. Mater.* **2017**, *29*, 1606964.

[13] W. Liao, D. Zhao, Y. Yu, C. R. Grice, C. Wang, A. J. Cimaroli, P. Schulz, W. Meng, K. Zhu, R. Xiong, Y. Yan, *Adv. Mater.* **2016**, *28*, 9333.

[14] S. J. Lee, S. S. Shin, Y. C. Kim, D. Kim, T. K. Ahn, J. H. Noh, J. Seo, S. I. Seok, *J. Am. Chem. Soc.* **2016**, *138*, 3974.

[15] Y. Liao, H. Liu, W. Zhou, D. Yang, Y. Shang, Z. Shi, B. Li, X. Jiang, L. Zhang, L. N. Quan, R. Quintero-Bermudez, B. R. Sutherland, Q. Mi, E. H. Sargent, Z. Ning, *J. Am. Chem. Soc.* **2017**, *139*, 6693.

[16] S. Shao, J. Liu, G. Portale, H. Fang, G. R. Blake, G. H. t. Brink, L. J. A. Koster, M. A. Loi, *Adv. Energy Mater.* **2018**, *8*, 1702019.

[17] C. Ran, J. Xi, W. Gao, F. Yuan, T. Lei, B. Jiao, X. Hou, Z. Wu, *ACS Energy Lett.* **2018**, *3*, 713.
